# Updates in Arrhythmia Management in Adult Congenital Heart Disease

**DOI:** 10.3390/jcm13154314

**Published:** 2024-07-24

**Authors:** Adam J. Small, Matthew Dai, Dan G. Halpern, Reina Bianca Tan

**Affiliations:** Medicine NYU Grossman School of Medicine, 530 First Ave, HCC 5, New York, NY 10016, USA; matthew.dai@nyulangone.org (M.D.); dan.halpern@nyulangone.org (D.G.H.); reina.tan@nyulangone.org (R.B.T.)

**Keywords:** arrhythmia, congenital heart disease, atrial flutter, atrial fibrillation, ventricular tachycardia, pacing, heart block

## Abstract

Arrhythmias are highly prevalent in adults with congenital heart disease. For the clinician caring for this population, an understanding of pathophysiology, diagnosis, and management of arrhythmia is essential. Herein we review the latest updates in diagnostics and treatment of tachyarrhythmias and bradyarrhythmias, all in the context of congenital anatomy, hemodynamics, and standard invasive palliations for congenital heart disease.

## 1. Introduction

Arrhythmias are highly prevalent in adults with congenital heart disease (CHD), occurring in nearly 20% of patients [1]. Arrhythmias are one of the most common reasons for hospital admission for adults with CHD across all levels of lesion complexity [2], and are risk factors for excess mortality in this population [3,4]. For the clinician caring for adults with CHD, an understanding of pathophysiology, diagnosis, and management of arrhythmia is essential. Table S1 provides a summary of arrhythmias in adults with CHD.

### Initial Evaluation of Arrhythmia

Evaluation of arrhythmia in CHD requires knowledge of anatomy, past interventions, and hemodynamics. Certain lesions are associated with specific arrhythmia mechanisms, like accessory pathways in the Ebstein anomaly or sinus node dysfunction after an atrial switch operation. The differential diagnosis should consider epidemiologic likelihood.

In addition to electrocardiography, a hemodynamic assessment should occur in all patients with CHD and suspected or confirmed arrhythmia. This includes history and physical examination, as well as transthoracic echocardiography in most cases. Further evaluations like transesophageal echocardiography, MRI, or hemodynamic catheterization may be added when needed to define the severity of a lesion. Ventricular dysfunction, cyanosis, obstructive lesions, and valvular regurgitation could all contribute to the development of arrhythmia and may need to be addressed in parallel with the arrhythmia per se. Coronary evaluation should be considered in patients over 40 years of age with ventricular arrhythmia, or in those with ventricular arrhythmia and lesions predisposing to coronary disease like arterial switch operation. Exercise testing can be a useful adjunct to provoke arrhythmia and to give general prognostic data [5].

After initial evaluation and electrocardiogram, patients with mild symptoms and low anatomic complexity may be appropriate candidates for outpatient ambulatory rhythm monitoring. A low threshold should be maintained for the invasive electrophysiology study of those with syncope, aborted sudden death, or high anatomic complexity. A patient with a univentricular heart, for example, may tolerate arrhythmia poorly and experience clinical heart failure if untreated for a few days [5].

## 2. Tachyarrhythmias

### 2.1. Overview of Supraventricular Tachycardias

Atrial arrhythmias (AAs) are highly prevalent in adults with congenital heart disease (CHD), occurring in as many as 15% [6]. Multiple studies have demonstrated correlation between AA and major morbidity, including heart failure and stroke, as well as death, with mortality risk at least two times higher among those with CHD and atrial arrhythmias compared to those without [6,7]. Unsurprisingly, AAs also significantly affect quality of life and perceived health status among patients with CHD [8].

Development of AA is associated with CHD complexity, as well as ventricular and valvular dysfunction [9]. Chronic right atrial overload is thought to result in structural remodeling including fibrosis, larger myocyte diameter, and longer capillary distance, all of which facilitate arrhythmia [10]. Certain lesions including Ebstein anomaly and tricuspid atresia are particularly prone to AA, perhaps due to both the congenital substrate and the chronic right-sided overload [11]. The progressive nature of fibrosis helps explain how the prevalence of permanent, rather than paroxysmal, AA increases with age [12].

### 2.2. Atrial Flutter and Atrial Fibrillation

Intra-atrial re-entry circuits propagate around barriers to electrical conduction such as surgical suture lines. Even an unrepaired atrial septal defect has been shown to facilitate atrial flutter by acting as an electrical barrier [13]. In the CHD population as a whole, intra-atrial re-entry and cavotricuspid-related atrial flutter are the most common AA mechanisms [14,15]. A history of atriotomy does not necessarily indicate the scar itself will be the relevant electrical barrier. Isthmus-dependent atrial flutter, rather than incisional flutter, is still the most common intra-atrial re-entry mechanism in this setting, and scar location does not reliably predict flutter mechanism [16].

The incidence of atrial fibrillation increases with age and becomes the most prevalent AA mechanism in adults with CHD over 50 years of age [17]. For the adult with CHD, the onset of atrial fibrillation is thought to result from age-related atrial remodeling as well as from scars from prior atriotomies and hemodynamic disturbances [18]. Importantly, heart failure commonly coincides with atrial fibrillation in adults with CHD [19]. That certain heart failure therapies such as dapagliflozin offer benefits for prevention atrial fibrillation [20] suggests that atrial fibrillation and heart failure should be addressed together and share some pathophysiologic pathways.

### 2.3. Focal Atrial Tachycardia

Focal atrial tachycardia may occur across all age groups and complexities of CHD, although it accounts for a smaller proportion of AAs among CHD patients over age 50, in whom atrial fibrillation is more prevalent [17]. Focal atrial tachycardia may originate from areas of abnormal conduction, the same areas which serve as pathways for slow conduction in large re-entrant circuits (atrial flutter) [15].

### 2.4. Atrioventricular Nodal Re-Entrant Tachycardia and Accessory Atrioventricular Conduction

Atrioventricular nodal re-entrant tachycardia (AVNRT) in a patient with congenital heart disease may pose unique treatment challenges due to an unusual AV node location and lack of usual anatomic landmarks and access routes. In congenitally corrected transposition of the great arteries, for example, the AV node is often found superiorly, medial to the right atrial appendage. In a subset of patients with heterotaxy syndrome, the AV node is duplicated, a phenomenon referred to as “twin AV node” physiology. In spite of these challenges, high rates of acute success > 90% for AVNRT ablation have been reported in CHD. Success rates are lower in complex CHD compared to simple CHD, and rates of atrioventricular block and pacing after ablation also correlate with higher complexity CHD [21,22].

For patients with accessory atrioventricular conduction pathways and atrioventricular re-entrant tachycardia (AVRT)—an arrhythmia classically associated with Ebstein anomaly but also occasionally seen in other lesions including single ventricle congenital heart disease—multiple pathways can be found in a subset of these patients. The presence of multiple atrioventricular conduction pathways is a risk factor for arrhythmia recurrence after ablation [23,24].

### 2.5. Management of Atrial Arrhythmias

#### 2.5.1. Atrial Anti-Tachycardia Pacing

If an implanted device is present, atrial anti-tachycardia pacing (ATP) can reduce the need for DCCV and in some cases delay or avert the need for ablation. One series reported 72% efficacy of ATP in patients with CHD [25].

#### 2.5.2. Direct-Current Cardioversion

For many CHD patients, direct-current cardioversion (DCCV) is a necessary and/or reasonable treatment for AA. DCCV is acutely effective in most cases, with comparable success in patients with CHD to those without, excepting patients with Fontan circulation, who are more likely to have DCCV failure and AA recurrence [26]. At three months of follow-up, though, adults with CHD have appreciable recurrence rates, as high as 46% [27]. For this reason, catheter ablation is often pursued.

#### 2.5.3. Catheter Ablation

Compared with DCCV, catheter ablation reduces the need for repeat DCCV [28]. Additionally, since antiarrhythmic options are often limited (this topic discussed later in the review), practitioners frequently refer early for catheter ablation. In spite of its advantages, however, the risk of AA recurrence after ablation is significant with rates as high as 40–50% at 3–4 years of follow-up [14,28,29,30]. As with DCCV, those with Fontan palliation fare worse after ablation, with lower procedural success and earlier recurrence of AA [14,30]. Previous atrial fibrillation and diagnosis of transposition of the great arteries (likely relating to the atrial switch repair technique) are also factors associated with procedural failure [30,31].

For patients with moderate or severe-complexity CHD, catheter ablation performed by an electrophysiologist specializing in CHD results in fewer repeat procedures compared with ablation performed by a non-CHD specialist [32]. Structural defects in the CHD population, as well as extensive atrial scarring, introduce unique challenges in the diagnosis and ablation of arrhythmia [33,34]. Anatomic and surgical electrical obstacles both may be encountered, and each has different properties like pathway length and conduction velocity [35]; the presence of multiple pathways is frequent and reduces the probability of procedural success [36]. When programmed atrial stimulation reveals new nonclinical AAs, targeting those pathways may be associated with lower rates of AA recurrence [37].

Given the complexities of the catheter ablation procedure in CHD, a multimodality imaging evaluation prior to ablation is essential [38]. Pre-procedural images must be merged with the electroanatomic map. Intracardiac echocardiography (ICE) is also an important adjunct. Use of ICE has been correlated with procedural success, as well as lower radiation exposure and procedure duration [39]. While arrhythmia circuits may be found in predictable anatomic landmarks, ultra-high-density activation mapping may assist in defining arrhythmia circuits in complex anatomy [40].

#### 2.5.4. Arrhythmia Surgery

For those undergoing open-heart surgery with high AA risk or difficult-to-manage AAs, arrhythmia surgery can be considered. The cut-and-sew Cox Maze III procedure has been associated with high rates of freedom from atrial fibrillation, exceeding 90% in some studies [41]. A right-sided maze procedure can be effective for atrial flutter in patients with high-risk anatomy like an Ebstein anomaly. One series reported 82% 1-year freedom from arrhythmia and 67% 5-year freedom in CHD patients with pre-existing AAs undergoing surgical treatment [42].

### 2.6. Ventricular Tachycardia

Much like with AA, ventricular tachyarrhythmias in patients with CHD can occur as a consequence of both the congenital defects themselves, as well as the surgical repair. Generally, ventricular arrhythmias in the latter category are more common and result of discrete substrates created by the operative repair of CHD, typically manifesting as monomorphic ventricular tachycardia (VT). In contrast, ventricular arrhythmias related to the underlying CHD itself tend to have more diffuse and poorly defined substrates, resulting in a wide range of arrhythmias from monomorphic VT, to polymorphic VT, to ventricular fibrillation (VF).

The archetype for VT in CHD has long been tetralogy of Fallot (TOF), in which there is robust clinical experience and data on the mechanisms of VT. Much of the approach to monomorphic VT in other forms of CHD is extrapolated from experience with TOF patients. The prevalence of sustained VT in repaired TOF is estimated to be between 3 and 14%, and accounts for the majority of the 0.2% annual risk of sudden cardiac death (SCD) [43,44,45]. The majority of these arrhythmias are macro-re-entrant monomorphic VTs that utilize discrete and well-defined substrates created following surgical repair. Specifically, the ventricular septal defect (VSD) patch and right ventricular outflow tract (RVOT) repair, often accompanied by a ventriculotomy in earlier techniques, result in scar formation and regions of slow conduction that create four critical isthmuses. These four isthmuses are located in the following: (1) between the RVOT patch/incision and tricuspid annulus, (2) between the RVOT patch/incision and pulmonary annulus, (3) between the VSD patch and pulmonary annulus, and (4) between the VSD patch and tricuspid annulus [46]. The presence (or absence) of the isthmuses is dependent on the surgical repair technique; isthmus 2 is absent in up to 75% of TOF patients, particularly in those with transannular patches, and isthmus 4 is absent in up to 87% of patients [47]. Isthmus 3, between the VSD patch and pulmonary annulus, is the most frequently present isthmus (>90% of patients) and is also the most frequently implicated in VT circuits [45,48]. This is likely due to its position in the conal septum, which connects the anterior RV free wall to the posterior RV septum and allows for formation of a macro-re-entrant VT circuit within the RV, similar to the role of the cavotricuspid isthmus in propagating typical atrial flutter within the right atrium.

Given the macro-re-entrant mechanism with well-defined isthmuses, radiofrequency catheter ablation has emerged as an effective treatment option for monomorphic VT in TOF and related CHDs, with acute procedural success rates of up to 86% [49]. However, it is important to note that although ablation has been shown to decrease rates of recurrent VT and implantable cardioverter-defibrillator (ICD) shocks, it has not been shown to attenuate the risk of SCD [45]. Thus, it serves as an adjunctive, not replacement, therapy to ICDs. The 2014 Pediatric and Congenital Electrophysiology Society and Heart Rhythm Society guidelines gave a Class I indication for catheter ablation in CHD patients with recurrent VT [5]. Ablation strategies depend in part on the clinical tolerability of the ventricular arrhythmia. Three-dimensional electroanatomic mapping is used to identify regions of scarring and the critical isthmuses. Activation mapping and entrainment maneuvers can be performed for slower and hemodynamically tolerated VTs, allowing for mapping of the precise tachycardia circuit. If the VT is not hemodynamically tolerated, ablation typically focuses on substrate modification, particularly of isthmus 3 in the conal septum, with the goal of creating a bidirectional block across the isthmus. Ablation success can be limited by the thickness of the conal septum, which impairs the ability to create duration transmural lesions, along with its proximity to the His-Purkinje system, which raises the risk of atrioventricular block. Sometimes, left-sided ablation of the septum via retrograde aortic access is required. In addition, catheter stability in the RVOT can be compromised by pulmonic regurgitation, which is often significant in repaired TOF patients. In cases where catheter ablation is not technically feasible or successful, surgical cryoablation can also be considered as a viable alternative, particularly if the patient is undergoing open heart surgery for other reasons (e.g., pulmonic regurgitation).

The advent of transcatheter pulmonic valve replacement (PVR), the most common reintervention in TOF, has presented a unique dynamic with catheter ablation. These prosthetic valves cover much of the RVOT including the arrhythmogenic isthmus 3 and conal septum, rendering future ablations in this area technically difficult and high-risk. The role of routine pre-PVR electrophysiologic (EP) studies and prophylactic VT substrate ablation is an ongoing area of research, with initial data demonstrating high rates of inducible VT, which has been shown to correlate with the future development of sustained VT and SCD [50,51]. Interestingly, PVR itself has also been shown to decrease the incidence of ventricular arrhythmias, likely related to improvements in RV hemodynamics and reverse remodeling of the associated RV myopathy [12].

Monomorphic VT in other repaired congenital defects besides TOF, such as in pulmonary stenosis and atresia, VSDs, double outlet right ventricle, and double outlet left ventricle, result from similar macro-re-entrant pathophysiologic mechanisms [45]. The location of the critical isthmuses varies depending on the lesion and surgical repair. However, given the importance of the RVOT and conal septum in propagating macro-re-entry, congenital lesions involving repairs in this location tend to confer the highest risk of monomorphic VT.

In contrast to VT enabled by surgical scars, some ventricular arrhythmias in CHD can occur as a result of the congenital defect itself. Typically, this is due to progression of an associated cardiomyopathy. The most commonly implicated CHD lesions include congenital aortic stenosis, Ebstein anomaly, Eisenmenger syndrome, and lesions resulting in a systemic RV-like congenitally corrected transposition of the great arteries (cc-TGA) or D-TGA following an atrial switch repair [43,45]. In these conditions, chronic progressive myopathies of the left and/or right ventricle lead to diffuse myocardial fibrosis and scarring, which serve as the nidus for a variety of ventricular tachyarrhythmias, ranging from focal and micro-re-entrant monomorphic VT to polymorphic VT to VF. Given the diffuse substrate involved, these ventricular arrhythmias are typically less amenable to catheter ablation, and management is focused on risk stratification for the prevention of SCD. The risk of SCD in congenital heart disease, including the role of ICDs, will be discussed further in a separate section.

### 2.7. Antiarrhythmic Drug Therapy

In the current era, antiarrhythmic drug therapy is typically used together with catheter ablation procedures, with the plan of care orchestrated and executed by electrophysiologists familiar with the anatomy and palliation of CHD. Pharmacologic therapy may be challenging in the CHD patient due to co-morbidities such as baseline conduction abnormalities, ventricular dysfunction, and liver and renal dysfunction, as well as child-bearing considerations. For many, antiarrhythmic options are limited. In addition, since multiple antiarrhythmic drugs are associated with bradycardia, it is important to evaluate the baseline conduction system prior to initiation of an agent. In some cases where use of a specific agent is recommended and symptomatic bradycardia is anticipated as a result, permanent pacing may be required.

Atrial tachyarrhythmia (e.g., IART) in simple and moderate-complexity lesions (e.g., ASD, VSD, Ebstein Anomaly, TOF) with normal ventricular function may be treated with class IC antiarrhythmic agents such as flecainide and propafenone. Class IC agents require a concomitant AV nodal blocking agent to prevent the organization of atrial fibrillation into a rapidly conducted 1:1 atrial flutter. Class IC antiarrhythmic drugs are not to be used in the setting of hepatic dysfunction, prolonged PR interval (>250 msec), coronary artery disease, or moderately to severely depressed systolic ventricular function.

In complex lesions (e.g., cyanotic disease or Fontan palliation) or cases with ventricular dysfunction, class III antiarrhythmics may be used, including amiodarone, sotalol, or dofetilide. Class III antiarrhythmic agents prolong the QTc interval, which may be challenging to calculate in the CHD patient due to a baseline bundle branch block or interventricular conduction delay causing a wide QRS complex. One strategy to account for this conduction delay is to use the following equation: QTc (corrected) = (QT − (QRS − 120))/RR^0.5^). Among the class III antiarrhythmic drugs, amiodarone is the most effective agent for atrial tachyarrhythmia; however, its use is cautioned in patients with cyanotic heart disease, prolonged QTc, low body mass index, or hepatic, pulmonary, or thyroid disease due to its broad toxicity profile [5]. Sotalol is considered a second-line therapy with mixed data regarding its safety.

Ventricular arrhythmias are primarily treated with beta-blockers and class III antiarrhythmic drugs, with amiodarone being the most commonly used agent. Drug therapy is used in combination with radiofrequency ablation for scar-related ventricular tachycardia (VT) and consideration of an implantable cardioverter-defibrillator (ICD) device when concern remains about the potential for syncope or sudden death. Surgical repair of hemodynamic lesions (e.g., pulmonary valve replacement in TOF) may improve the arrhythmic burden but may not eliminate the risk. Acute care for VT should follow practice guidelines as set forth by the American Heart Association. For hemodynamically stable VTs, amiodarone, procainamide and lidocaine are suggested. Procainamide is effective for macro-re-entrant monomorphic VTs whereas lidocaine is more effective for ischemic VTs [5].

### 2.8. Anticoagulation

Lower thresholds are used for anticoagulation (AC) in the ACHD population owing to their inherent structural abnormalities which are thought to promote thrombus formation. The application of the CHA2DS2-VASc scores is challenging since ACHD patients tend to be younger and lack the traditional cardiovascular risk factors—yet they may still have co-morbidities that predispose to thrombosis such as cyanosis or Fontan palliation. In common ACHD practice, AC is prescribed most to patients with atrial fibrillation, CHADS2-VASc score ≥ 1, and Fontan circulation [52]. Decision-making with simple lesions is based on the known established scores for stroke risk (e.g., CHA2DS2-VASc) and bleeding risk (e.g., HAS-BLED) [5]. Long-term full AC is recommended for moderate or complex lesions with sustained or recurrent tachyarrhythmia [5]. Non-vitamin K oral anti-coagulants (NOACs) are emerging as safe and effective for use among ACHD patients. A large review found low rates of complications with these agents, with <1% thromboembolism and 1.85% major bleeding [53]. Still, further data are needed as use of these agents increases. A study of ACHD patients from a large German registry found increased risk of mortality and bleeding with NOACs compared to vitamin K antagonists, even when adjusted for patient characteristics [54].

### 2.9. Arrhythmias and Transplantation

In rare cases, refractory arrhythmia may be an indication for cardiac transplantation. Among congenital heart lesions, this scenario may be most common in the univentricular heart after Fontan palliation. Given the risk of Fontan conversion surgery and the high recurrence rate of arrhythmia, transplant may be considered at the time Fontan conversion is considered [55,56]. Even in advanced stages, catheter ablation may help palliate arrhythmia or even delay the need for transplantation [57].

### 2.10. Technological Advances

In recent decades, significant advances have taken place for the management of arrhythmia in patients with CHD. For patients seen in the office, current ambulatory rhythm monitors capture more granular data for longer periods of time and are significantly less cumbersome than older monitors. For rarer arrhythmias, implantable loop recorders can obtain years of data, which can be useful either for symptom–rhythm correlation or for evaluation of cryptogenic stroke. Even consumer-wearable EKG devices built into smart watches and stand-alone devices such as the Kardia mobile device (AliveCor, Mountain View, CA) can be useful tools for diagnosis and management of arrhythmias in the outpatient setting [58].

For patients requiring ablation, mapping systems and ablation techniques have both advanced significantly in recent decades. Improvements in electroanatomic mapping systems, as well as use of intracardiac echocardiography and transesophageal echocardiography, have reduced the amount of radiation necessary for electrophysiologic procedures, so an increasing number of cases can be done with zero fluoroscopy. Using a remote magnetic navigation technique, soft and flexible catheters can be remotely steered to difficult-to-reach locations such as the pulmonary venous atria to create three-dimensional electrical maps, with no radiation and very low perforation risk. This is especially beneficial for the CHD population, who are younger, may be or become pregnant, and may need to undergo multiple procedures [58].

Cryoablation, another technique gaining widespread use in recent decades, also offers advantages to the CHD patient. Different from radiofrequency ablation, cryoablation causes reversible effects before permanent tissue destruction. The catheter tip also adheres to the endocardial surface, enhancing catheter stability. For arrhythmias near the sinus or atrioventricular node, or for cases where location of the conduction system is challenging, these are particularly useful traits [58]. Cryoablation has gained widespread use in treatment of atrial fibrillation, which is expected to become increasingly prevalent in an aging CHD population [17].

Pulsed field ablation (PFA) is a non-thermal method of ablation which uses high-voltage electrical fields to disrupt myocardial cell walls and cause tissue necrosis. PFA uses ultrarapid electrical pulses to ablate myocardial tissue while sparing surrounding tissue preferentially, thus minimizing complications. Its use has been reported in adults with congenital heart disease and AA, and effective ablative lesions have been generated in thickened atrial myocardium using this technique [59]. Novel pulsed field ablation systems may offer unique procedural approaches by combining pulsed field ablation with high-density mapping and radiofrequency, all in the same procedure.

## 3. Bradyarrhythmias

### 3.1. Epidemiology and Anatomy of Bradyarrhythmia in CHD

#### 3.1.1. Sinus Node Disease

The sino-atrial node (SAN) is typically located at the junction of the crista terminalis of the right atrium and the os of the superior vena cava. In congenital heart abnormalities that lack right atrial anatomy such as left atrial isomerism (heterotaxy), the SAN may be absent. Alternatively, it may be aberrantly located anteriorly and dysfunctional [60]. In cases of right atrial isomerism, it may be duplicated [5,43,61]. Overall, though, the main causes of SAN dysfunction in CHD relate to surgical interventions and scarring that involve the SAN area. The Mustard atrial switch operation for d-transposition of the great arteries (d-TGA) is a notable intervention that has been shown to result in SAN dysfunction, with more than one third of patients requiring permanent pacing [62,63]. Other operations associated with SAN dysfunction include superior sinus-venosus defect repair, Ebstein Anomaly repair, tetralogy of Fallot (TOF) repair, and Fontan palliation. Chronotropic insufficiency, which may accompany SAN dysfunction, is associated with limiting symptoms during exertion, as well as the development of atrial tachyarrhythmia [5,61]. Acquired conditions may also present with bradycardia, including obstructive sleep apnea, athleticism, hypothyroidism, cardiomyopathies, increased intracranial pressure, and some unique infections such as Lyme disease.

#### 3.1.2. Atrio-Ventricular Node Disease

The AV node (AVN) is typically located within the triangle of Koch in the posterior-inferior atrial septal area, with the His bundle extending from it. The triangle of Koch is classically delineated by the septal leaflet of the tricuspid valve, the ligament of Todaro and the coronary sinus os, but its location may vary in certain congenital heart lesions. In congenitally corrected transposition of the great arteries (cc-TGA), for example, the AVN is displaced anteriorly and superiorly. This lesion is associated with 2% yearly risk of complete heart block, and risk is considered particularly elevated in cases with surgical correction of a ventricular septal defect (VSD) or tricuspid valve intervention [61]. In cases of atrioventricular canal defects, the AVN and the His bundle are posteriorly located with hypoplasia of the anterior fascicle, causing a first-degree AV block and left axis deviation on ECG. This lesion is also prone to spontaneous heart block [62]. Acquired heart block may be seen as a post-operative complication (1–3% of CHD surgery) after surgery involving the left ventricular outflow tract (e.g., aortic replacements, septal myectomy or subaortic stenosis resection, VSD repair), a displaced conduction system (cc-TGA, AV canal defects), or a mitral or tricuspid valve intervention [5,64]. In cases of post-operative heart block, a waiting period of 7–10 days is advised prior to permanent pacing, since transient node edema may recover in half of patients [64].

### 3.2. Cardiac Pacing

#### 3.2.1. Pacemaker Implantation in Adults with Congenital Heart Disease

Cardiac implantable electronic devices (CIEDs) are increasingly used in patients with congenital heart disease. Indications for anti-bradycardia device therapy are similar to those for non-CHD patients, such as sinus node dysfunction (SND) or atrioventricular block (AVB). In adults with congenital heart disease, CIED implantation is highly complex, owing to issues with venous access to chambers, venous obstruction, coronary sinus location, venous anomalies, dilated chambers, comorbidities, high capture thresholds or poor sensing due to fibrosis, oversensing due to chamber hypertrophy, and valve related issues.

#### 3.2.2. Indications

The 2014 Pediatric and Congenital Electrophysiology Society (PACES) and Heart Rhythm Society (HRS) expert consensus statement on the recognition and management of arrhythmias in ACHD gives guidance to indications for device implantation and makes specific recommendations with regards to anatomy, surgical repair, and pacing mode [5]. The strongest indications for pacing are for symptomatic bradycardia, AV block with wide-QRS escape and/or ventricular dysfunction, and postoperative high-degree AV block that is not expected to resolve. Bifascicular block and first-degree AV block are not considered appropriate indications for pacing on their own, and transvenous systems are generally avoided in the presence of an intracardiac shunt. Regarding cardiac resynchronization therapy, the strongest indication exists for systemic LVEF < 35%, QRS > 150 ms, left bundle-branch morphology, and symptoms. A narrow QRS complex and a highly limited life expectancy < 1 year are considered contraindications to resynchronization therapy.

#### 3.2.3. Technical Considerations

Standard transvenous systems may not be feasible due to venous anatomy or contraindicated due to the presence of a significant intracardiac shunting, thus creating a risk for thromboembolism due to intravascular leads. The presence of intracardiac shunt or single ventricle physiology are generally contraindications to transvenous pacemaker implantation due to a two-fold greater risk of systemic thromboembolism [65]. Epicardial implantation is preferred in certain circumstances; however, the surgery is more involved, and long-term lead performance may be inferior to transvenous systems [66].

### 3.3. Transvenous Pacemaker

Careful planning is paramount when considering a transvenous pacemaker implant. A full understanding of the patient’s anatomic defect, including any known or anticipated associated anomalies, careful review of all prior surgical and device procedures (including venograms from prior cardiac catheterization or device procedures) as well as advanced imaging (CT or MRI) are essential. It is important to note the presence of synthetic septal patches, atrial baffles, conduits, obstructed venous channels, and persistent left superior vena cava (SVC).

Patients post-Fontan palliation lack standard venous connection to the ventricle. Transvenous atrial lead placement may be possible in the atrio-pulmonary Fontan and lateral tunnel Fontan modifications as there is access to atrial myocardium [67]. In extracardiac or intra-extracardiac Fontan, placement of an atrial lead can be challenging; there have been several reports of implant within the pulmonary venous atria via a puncture through the pulmonary artery into the roof of the common atrium [68,69]. The risk of thromboembolic events with leads in the systemic circulation should be strongly considered before considering this option [65,70].

In patients with an atrial switch (Mustard or Senning), the presence of baffle stenosis or leaks should be evaluated. Stenting the baffle prior to lead implantation should be considered due to the high incidence of baffle occlusion after lead placement.

### 3.4. Epicardial Pacemaker

The advantages of epicardial systems include placement ability to implant during concomitant surgery, avoidance of venous access, and lower thromboembolic risk. However, epicardial systems require a sternotomy/lateral thoracotomy in a patient not getting concomitant surgery or intrathoracic surgery for future lead revisions, leading to higher incidence of lead failures and longer recovery times [71,72,73]. The optimal site of epicardial ventricular lead placement is the LV apex or mid lateral wall [74,75]. RV apex and anterior free wall lead placement have been shown to result in the long-term risk of LV remodeling, dyssynchrony, and dysfunction [76,77].

### 3.5. Leadless Pacemaker

For patients with challenging vascular access, tricuspid valve disease, intracardiac shunt, and/or high risk of device infection, a transcatheter or leadless pacemaker may be considered to avoid common complications or contraindications to transvenous devices. Leadless pacemaker implantation has been reported in adults with CHD [78]. This procedure is a useful option for some CHD patients, given the frequency of transvenous pacer complications and contraindications in this population. Potential limitations of transcatheter pacemaker implantations include a large-caliber delivery system, exclusion of pulmonary hypertension from initial trials, and lack of atrial sensing or pacing. Some of these limitations are being addressed in newer iterations of the leadless pacemaker including dual chamber leadless pacemaker systems.

#### Heart Failure and Pacing

Ventricular pacing has been associated with pacing-induced cardiomyopathy. Risk factors include a longer intrinsic or paced QRS duration, depressed ventricular function at baseline and higher pacing burden [79,80]. In ACHD patients, the incidence of pacing induced cardiomyopathy is 25% and increases to 47% if the pacing burden is >70% [81]. Single-site sub-pulmonary ventricular pacing is an independent predictor of systemic RV dysfunction in ccTGA [82].

### 3.6. Cardiac Resynchronization Therapy

Pacing-induced cardiomyopathy can be prevented or reversed with cardiac resynchronization therapy (CRT), whereby an additional lead is placed in the coronary venous system. CRT is an established treatment modality for systolic heart failure associated with left ventricular electromechanical dyssynchrony. Despite nonstandard indications and anatomic constraints on lead placement in CHD patients, results have been promising [83,84,85]. Improvement in ventricular synchrony, New York Heart Association (NYHA) functional class status, and ejection fraction have been reported among patients with TOF and single ventricular physiology. CRT in CHD often requires epicardial pacing lead placement due to limited access to a ventricular chamber (as in patients with Fontan), abnormal coronary venous anatomy, or exclusion of the coronary sinus from the right heart by surgical patches. A hybrid approach should be also considered with transvenous lead insertion in the subpulmonary LV and epicardial pacing of the systemic RV in atrial switch.

In structurally normal hearts, the strongest predictor for response to CRT include LBBB morphology and QRS duration greater than or equal to 150 ms. In CHD, surface electrocardiogram morphology is frequently not a strong indicator of the exact underlying cause of electrical dyssynchrony, such as a bundle branch block. Cardiac morphology may also play an important role in CRT response; however, results have been conflicting as CRT responses have been observed in all groups depending on the study and it remains unclear if a particular cardiac morphology precludes CRT application [83,84,85,86,87,88]. Assessment of response to CRT in CHD is highly challenging in this heterogenous population, and there is no single parameter accepted as a marker of response.

### 3.7. Conduction System Pacing

Cardiac conduction system pacing (CSP) was first described in 2000 and has been increasingly accepted and sometimes the preferred method to achieve cardiac resynchronization. Methods include His bundle pacing (HSP) or left bundle branch pacing (LBBP). CSP has been proven to be safe and feasible with encouraging outcomes in non-CHD patients [89,90]. In ACHD patients, potential barriers to CSP include the presence of surgical scarring near the conduction tissues, and the potential impact of variations in conduction system anatomy in CHD on CSP performance remain unknown. However, studies have shown that despite anatomical variations and surgical repair in the vicinity of the conduction system, CSP may be both feasible and durable in CHD. CSP has resulted in greater QRS narrowing and at least equivalent improvement in LVEF as compared with conventional CRT in CHD [91]. In CCTGA, HBP has had a favorable clinical response with excellent lead characteristics over time likely due to the course of the conduction system making it particularly amenable to CSP [92,93].

## 4. Conclusions

The management of arrhythmia requires a nuanced understanding of a patient’s anatomy, hemodynamics, and past palliations. Contemporary tools have alleviated symptoms and prolonged life in many patients, and technology promises to evolve even further. Effective management of the CHD patient with arrhythmia should ideally be undertaken with a multidisciplinary team.

## Supplementary Materials

The following supporting information can be downloaded at: https://www.mdpi.com/article/10.3390/jcm13154314/s1, Table S1: Summary of Arrhythmias in Adult Congenital Heart Disease.
