# Supplementary material for: Updates in Arrhythmia Management in Adult Congenital Heart Disease
Source: J Clin Med. 2024 Jul 24;13(15):4314. doi: 10.3390/jcm13154314 (PMC11312906; doi:10.3390/jcm13154314)
Supplement: Supplementary file 1 [file jcm-13-04314-s001.zip › jcm-3083437-supplementary.pdf]

Table S1. Summary of Arrhythmias in Adult Congenital Heart Disease.

Arrhythmia	Mechanism	Patients at Risk	Treatment*	Other Considerations
Atrial Flutter/inter-atrial re-entrant tachycardia	Macro-re-entry around anatomic barrier (e.g., surgical scar)	Univentricular heart, atrial switch operation, prior atriotomy<br><br>Most frequent atrial arrhythmia in CHD	Catheter ablation typically including cavotricuspid isthmus ablation;<br>surgical ablation in select cases.<br><br>Class IC antiarrhythmic agents may be used as adjunct to AV nodal blockers such as beta blockers, calcium channel blockers	Consider early DCCV in patients at high risk for developing heart failure such as single-ventricle, atrial switch<br><br>Atrial ATP can be useful adjunct in those with implantable devices
Focal atrial tachycardia	Circumscribed area of activation with centrifugal spread	Younger at presentation with more complex CHD	Catheter ablation;<br>surgical ablation in select cases.<br><br>Class IC antiarrhythmic agents may be used as adjunct to AV nodal blockers such as beta blockers, calcium channel blockers	
Atrial fibrillation	Multiple ectopic foci or single circuit re-entry triggering diffuse disorganized (fibrillatory) activity	Second most frequent atrial arrhythmia<br>In CHD	Antiarrhythmic drugs<br>Pulmonary vein isolation	Most frequent over 50 years of age
Atrioventricular re-entrant tachycardia	Accessory atrioventricular conduction	Ebstein, CCTGA	Accessory pathway ablation	
Atrioventricular nodal re-entrant tachycardia	Re-entrant circuit near AV node	Limited data on risk factors in CHD	Slow pathway modification	AV node anatomy is highly variable in congenital heart disease, making ablation more challenging
Ventricular arrhythmia	Monomorphic VT: Macro re-entry, as around a surgical scar<br><br>Polymorphic VT and ventricular fibrillation	TOF, right ventriculotomy<br><br>Hypertrophy, fibrosis, progressive right or left ventricular dilatation, and eventually heart failure.	Radiofrequency catheter ablation effective in TOF.<br>Surgical ablation in select cases<br><br>ICD	Surgery in the right ventricular outflow tract creates risk of monomorphic VT<br><br>Systemic RV, Ebstein anomaly and lesions involving a cardiomyopathy predispose to a variety of VT mechanisms

Sinus node dysfunction	Congenital or acquired; Transient edema of nodal tissue may be seen after surgery	D-transposition/Atrial switch, Left atrial isomerism, SVASD repair, Ebstein repair, Fontan Palliation	Transvenous or epicardial pacemaker	Intracardiac shunt or single ventricle physiology are generally considered contraindications to transvenous pacing
AV node dysfunction	Congenital or acquired; Transient edema of nodal tissue may be seen after surgery	Congenitally corrected transposition, atrioventricular septal defect, L-looped ventricles	Transvenous or epicardial pacemaker	May be seen postoperatively after surgery involving VSD repair, left ventricular outflow tract or atrioventricular valves.<br><br>Presence of intracardiac shunt is generally considered a contraindication to transvenous pacing
Electromechanical dys-synchrony	Pacing-induced cardiomyopathy	Ventricular dysfunction	Biventricular pacing, which may require surgical leads in certain CHD lesions<br><br>Conduction system pacing	Increased risk of pacing-induced cardiomyopathy with increased ventricular pacing burden, particularly > 70% pacing

*Initial evaluation of arrhythmia should always follow ACLS protocol as set forth by the American Heart Association
